# Influenza, Winter Olympiad, 2002

**DOI:** 10.3201/eid1201.050645

**Published:** 2006-01

**Authors:** Adi V. Gundlapalli, Michael A. Rubin, Matthew H. Samore, Bert Lopansri, Timothy Lahey, Heather L. McGuire, Kevin L. Winthrop, James J. Dunn, Stuart E. Willick, Randal L. Vosters, Joseph F. Waeckerle, Karen C. Carroll, Jack M. Gwaltney, Frederick G. Hayden, Mark R. Elstad, Merle A. Sande

**Affiliations:** * University of Utah School of Medicine, Salt Lake City, Utah, USA;; † Veterans Affairs Medical Center, Salt Lake City, Utah, USA;; ‡ Centers for Disease Control and Prevention, Atlanta, Georgia, USA;; § ARUP Laboratories, Inc., Salt Lake City, Utah, USA;; ¶ Lakeshore Medical Clinic, Milwaukee, Wisconsin, USA;; # Kansas City School of Medicine, Kansas City, Missouri, USA;; ** University of Virginia, Charlottesville, Virginia, USA

**Keywords:** Influenza, sports, Olympics, public health surveillance, prevention, prophylaxis, vaccination, dispatch

## Abstract

Prospective surveillance for influenza was performed during the 2002 Salt Lake City Winter Olympics. Oseltamivir was administered to patients with influenzalike illness and confirmed influenza, while their close contacts were given oseltamivir prophylactically. Influenza A/B was diagnosed in 36 of 188 patients, including 13 athletes. Prompt management limited the spread of this outbreak.

The Olympics are the quintessential organized sport where elite international athletes live in close quarters and compete in an intense environment. Upper respiratory illnesses occur frequently (*1*), and influenzalike illnesses (ILI) have been reported in previous Olympics (*2**–**6*). Prospective surveillance was conducted for influenza, with an emphasis on diagnosis, treatment, and prevention, during the 2002 Winter Olympic/Paralympic Games.

## The Study

This study was performed at the Olympic Village Polyclinic during the 2002 Winter Olympiad in Salt Lake City, Utah, USA, during February and March 2002. Athletes and nonathletes with upper/lower respiratory symptoms (with or without febrile/systemic illness) were screened for influenza by various modalities. Viral test results from the Polyclinic and public health reports of influenza in the local community were reviewed daily. Patients with ILI or confirmed influenza were offered treatment with oseltamivir; close contacts were offered prophylaxis (detailed methods available from corresponding author by email).

A total of 2,635 medical visits were recorded during the Games; patients with any respiratory symptom represented 12%. Of these, 188 satisfied the symptom criteria for the study (available from corresponding author) and were screened for influenza (Table 1). Influenza A was detected in 28 (15%) and influenza B in 8 (4%) patients (Table 2). Athletes comprised 36% of all influenza patients. Of the influenza A isolates, 8 were further analyzed and found to be consistent with the A/Sydney/97(H3N2) strain (represented in the 2001–2002 vaccine).

**Table 1:** Patients screened for influenza, 2002 Winter Olympic and Paralympic Games

Characteristic	Olympics, n (%)	Paralympics, n (%)
No. patients screened	156	32
No. countries represented	45	9
Age, y, mean (range)	34 (18–67)	37 (20–65)
Sex, male	98 (63)	15 (47)
History of influenza vaccination before arrival	37 (24)	7 (22)
Accreditation		
Organizing committee volunteers and staff	62 (40)	21 (65)
Athletes	41 (26)	5 (16)
Law enforcement personnel	29 (19)	1 (3)
Olympic family	24 (15)	5 (16)
Tests performed*		
Direct fluorescent antibody and viral culture	156 (100)	32 (100)
Rapid streptococcal antigen test	98 (63)	12 (38)
Rapid influenza test	141 (90)	19 (59)
Multiplex reverse transcription–polymerase chain reaction for respiratory viruses	33 (21)	4 (13)

*All patient specimens were screened for influenza by direct fluorescent antibody (DFA) and viral culture. Selected samples that were negative by DFA and viral culture were screened by reverse transcription–polymerase chain reaction for influenza. Rapid tests for influenza and streptococcal antigen were conducted on selected patients based on their symptoms and the clinician's discretion. Detailed methods are available from the corresponding author by email.

**Table 2:** Patients treated, 2002 Winter Olympic and Paralympic Games

Characteristic	Influenza A or B	Noninfluenza	Odds ratio (95% CI*), p adjusted
No. patients treated	36	152	
Accreditation (%)			
Organizing committee volunteers and staff	14 (39)	69 (45)	Reference
Athletes	13 (36)	33 (22)	2 (0.8–4.6), p = 0.1†
Law enforcement personnel	4 (11)	26 (17)	1.3 (0.2–2.5), p = 0.7
Olympic family	5 (14)	24 (16)	1 (0.3–3.2), p = 0.9
Age, y, mean (standard deviation [SD])	32 (10)	35 (13)	0.7 (0.4–1.0), p = 0.08‡
Sex, male %	78	56	5.5 (1.6–18.3), p = 0.006
Symptom duration, mean/median days (SD)	2.9/2 (3.5)	3.7/2 (5)	2.0 (0.7–5.8), p = 0.2§
History of influenza vaccination (%)	7 (19)	37 (24)	1.7 (0.5–1.6), p = 0.4
Temperature >37.8ºC (%)	14 (39)	7 (5)	13 (4.7–36), p<0.001
Symptoms (%)			
History of fever	22 (61)	40 (26)	1.2 (0.4–3.7), p = 0.8
Cough	33 (92)	90 (59)	25.7 (2.2–155), p<0.001
Chills	20 (56)	33 (22)	3.9 (1.2–12.8), p = 0.02
Myalgia	23 (64)	57 (38)	2.1 (0.7–6.4), p = 0.2
Sore throat	22 (61)	110 (72)	0.4 (0.1–1.4), p = 0.2

*CI, confidence interval. †Athletes are a significant group when influenza A cases alone are considered, odds ratio 3, 95% CI (1.1–7.5) p = 0.03. ‡Age as grouped by decade. §Symptom duration was grouped as <48 h or >48 h.

Patients with confirmed influenza (Table 2) were more likely to be male, have a temperature >37.8°C, and have a history of cough or chills. No significant differences were found in symptom duration or influenza vaccination status among those with and without influenza. Athletes were more likely to have a diagnosis of influenza A than other pooled groups of nonathletes (odds ratio [OR] 3, 95% confidence interval [CI] 1.1–7.5, p = 0.03).

Twenty-five of 188 patients who were screened by direct fluorescent-antibody assay (DFA) for influenza were positive. When the results were compared to viral culture alone, sensitivity was 70%, specificity was 99%, positive likelihood ratio was 54, and negative likelihood ratio was 0.3. Ten (6%) of the 160 who received a rapid influenza test had positive results. The sensitivity of the rapid test for diagnosing influenza (when compared to a confirmed diagnosis by viral culture, polymerase chain reaction, or DFA) was 17%, while the specificity was 97%. The positive likelihood ratio and negative likelihood ratio were 5.2 and 0.9.

The conventional syndromic definition of ILI (fever and either cough or sore throat) (*7*) had a low positive likelihood ratio of 2.7, negative likelihood ratio of 0.5, sensitivity of 67%, and specificity of 78% in predicting influenza. Overall, 23% of nonathletes and 18% of athletes screened reported influenza vaccination. Of those with confirmed influenza, vaccinees were likely to have lower fevers, although the results were not significant.

Physicians prescribed oseltamivir for 60 (32%) of 188 patients screened for influenza. Of the medicated patients, 40 (67%) were treated for ILI within 48 hours of symptom onset; influenza was confirmed in 21. Oseltamivir prophylaxis (for 5 days) was prescribed in 20 (33%) patients who had a history of contact with influenza patients; 1 case of influenza was confirmed in this group. All patients who received oseltamivir tolerated the medication well.

Three distinct clusters of ILI were identified during the Games. Cluster I consisted of 13 law enforcement personnel who worked and lived in close proximity. In early February, 3 members came to the clinic 4 days apart with ILI, and influenza A was diagnosed (2 cases by DFA, 1 by viral culture). Oseltamivir prophylaxis was promptly initiated in the remaining 10 asymptomatic members; the oseltamivir was well tolerated. No other cases of ILI were reported. The group was able to discharge its duties in the village.

Cluster II consisted of 12 members of a national team who had trained together at a common location 3 days before their arrival at the Olympic Village. Two days after they arrived, the index patient (unvaccinated for influenza) came to the clinic with ILI of 24 hours' duration and was given oseltamivir. Upon confirmation of influenza A by DFA, unvaccinated asymptomatic close contacts of the patients were offered oseltamivir prophylaxis; 8 of 11 accepted. In the next 4 days, 3 vaccinated teammates who had not received prophylaxis came to the clinic with ILI of 24 hours' duration. Treatment was initiated because of their close contact with the index patient. One patient was subsequently found to have influenza A by DFA. No further cases of ILI were reported. The team competed successfully in the sport and won several medals.

Cluster III consisted of 8 participants of 1 sport (which had 80 participants with common training venues) sought treatment at the Polyclinic within 9 days with respiratory symptoms (5 had ILI, 3 were afebrile). The 5 with ILI were treated with oseltamivir. Of the 3 afebrile participants, 2 were provided prophylaxis based on their contact history and symptoms. The third patient was not offered prophylaxis due to insufficient contact history. Influenza A was confirmed in 5 patients. No reports of ILI or confirmed influenza occurred among participants from this group after treatment/prophylaxis was initiated.

## Conclusions

This is the first systematic influenza study at any large international sports gathering and demonstrates the feasibility of managing influenza at such events. The intervention strategy integrated a policy of empiric treatment based on clinical data and viral testing with a public health surveillance approach, including daily review of all viral test results from the Polyclinic and reports of influenza in the community. Potential clusters of influenza were promptly identified, index patients were treated with oseltamivir, and contacts were given oseltamivir prophylaxis.

We examined several methods of detecting influenza from respiratory samples and found DFA testing to be the most useful surveillance tool in this setting. The sensitivity of rapid testing was low. This observation is consistent with the variability typically associated with rapid testing regarding patient age, duration of symptoms, type of kit, and timing of specimen acquisition (*7**–**9*).

A low rate of influenza immunization was noted among participants. The World Health Organization and others have suggested that vaccination is beneficial for athletes (*2**,**4**,**10**–**12*). Although this study was not designed to address the effectiveness of influenza vaccination, we support issuing a public health alert that encourages administering influenza vaccine to all athletes and staff before a large international event is staged.

Team physicians may not have reported all episodes of ILI to the Polyclinic, though this scenario is unlikely, given their frequent direct communication. Alternative strategies for influenza control, such as mass vaccination (*13*), were not examined in this study. Followup was not attempted since patients often dispersed to various international destinations after their events.

In summary, the surveillance and intervention strategy used in this study may serve as a model for mobilizing teams to provide health care to a large assembly of participants. Initiating empiric treatment for influenza based on clinical and epidemiologic data, combined with testing by DFA (with subsequent confirmation by viral culture), may be a prudent approach to influenza control in large gatherings. Close contacts of persons with positive DFA tests would then be candidates for prophylaxis. Similar approaches may enhance preparedness for public health threats and emerging respiratory pathogens such as avian influenza and agents of bioterrorism.
